# Molecular Organization in Exponentially Growing Multilayer Thin Films Assembled with Polyelectrolytes and Clay

**DOI:** 10.3390/polym14204333

**Published:** 2022-10-14

**Authors:** Biswa P. Das, Marina Tsianou

**Affiliations:** Department of Chemical and Biological Engineering, University at Buffalo, The State University of New York (SUNY), Buffalo, NY 14260-4200, USA

**Keywords:** polyelectrolytes, polyelectrolyte multilayers, thin films, polyelectrolyte complexes, layer-by-layer, clay

## Abstract

Multilayer thin film assembly by the layer-by-layer (LbL) technique offers an inexpensive and versatile route for the synthesis of functional nanomaterials. In the case of polymer-clay systems, however, the technique faces the challenges of low clay loading and lack of tunability of the film characteristics. This is addressed in the present work that achieves exponential growth in clay-containing polyelectrolyte films having high clay loading and tailored properties. Our approach involves the incorporation of a weak polyelectrolyte and a clay with relatively high charge density and small particle size. The system of investigation comprises poly(diallyldimethylammonium chloride) (PDDA) as the polycation and laponite clay and poly(acrylic acid) (PAA) or poly(sodium-4-styrene sulfonate) (PSS) as polyanions that are used alternately to create multilayers. Successful high clay loading and exponential growth were achieved by two different approaches of polyanion incorporation in the multilayers. A progressive increase in the degree of ionization of PAA was shown to contribute to the exponential growth. Our findings also include novel pathways to manipulate thickness, surface topography, and clay content. The strategy presented here can lead to novel approaches to fabricate tailor-made nanomaterials for distinct applications.

## 1. Introduction

Layer-by-layer (LbL) assembly, a technique initially utilized to form multilayers incorporating only polyelectrolytes, has been extensively researched in the last two decades to form unique multicomponent nanomaterials. The polyelectrolyte multilayers (PEMs) are a special case of polyelectrolyte complexes (PECs), which face technical challenges as viable engineering materials but possess tremendous research opportunities [1]. The technique has expanded to incorporate many other entities along with polyelectrolytes. Clay is one such component that has also been successfully LbL-assembled with polyelectrolytes to form advanced materials. Some of the most recent examples of the potential applications of the polymer-clay multilayers are as barrier thin films [2,3], anti-corrosion coatings [4,5], membranes with tailored nanochannels [6], phosphorescence thin films [7], waste treatment membranes [8], fire-retardant nanocomposites [9], super-strong chemically inert films [10], and in biomedical applications [11,12]. Clays are phyllosilicate minerals and are known for their exceptional mechanical and barrier properties. Moreover, they are inexpensive and abundantly available. In the earliest work on polymer-clay multilayers, Kleinfeld and Ferguson [13] showed that the disc-like clay particles could be arranged with consistent orientation and uniform spacing within the multilayer structure. Studies have shown that incorporation of clay results in improved mechanical properties in these films [14,15,16,17]. Clay is rigid (unlike polyelectrolytes, which are flexible) and has a high aspect ratio and hence has been reported to improve the barrier properties of the multilayered films [18,19]. Although rigid, platelets of clay have been reported to maintain the stretchability of multilayer films along with increasing the barrier properties [20]. Polymer-clay multilayers are superior to conventional nanocomposites formed by methods such as solution intercalation, in situ polymerization, melt processing, and sol-gel technique, as they enable greater and uniform clay loading and provide control over clay orientation [17,21,22].

The attractiveness of LbL assembly technique is that it is easy, inexpensive, and versatile. Although the focus has been on various potential applications, one fundamental unmet need is the speed of fabrication of clay-containing multilayers. Over the last decade, macroscopic film thickness in fewer deposition cycles has been achieved by an “exponential-growth” mechanism. It has been widely accepted that exponential growth is a result of “in-and-out diffusion” mechanism of weak polyelectrolytes [23,24,25]. The weak polyelectrolytes, during deposition, interact with the surface and also enter the bulk of the multilayer. These weak polyelectrolyte molecules that have entered the bulk also diffuse through the subsequent layers and reach the outermost surface during rinsing. This increases the net surface charge of the film, and as a result, a much higher amount of the subsequent polymer is deposited. In polymer-clay systems, impermeable clay particles theoretically act as impediment for “in-and-out diffusion” mechanism. Podsiadlo et al. [26] are the first to have reported exponential growth in multilayers incorporating clay, resulting in very thick layers for a relatively small number of deposition cycles. In their exponentially growing system, the clay content was very low (5–10 wt.%), and the difference in mechanical properties between clay-containing multilayers and only polymeric multilayers was minimal. Since then, only very few researchers have obtained exponential growth in a polymer-clay system [27,28], but they have not delved deep into the governing mechanism. Along with the low clay loading in the multilayers, the tunability of the clay composition and other physical parameters, such as thickness and surface morphology, are also open questions for practical applications of such systems. The scarcity of exponentially growing clay-containing systems is due to the lack of fundamental understanding of the physiochemical interactions in such systems.

Our aim is to investigate the structural organization, internal interactions, and composition of exponentially growing polymer-clay multilayers in order to better understand the different physicochemical aspects of exponential growth. The system of investigation comprised one polycation, poly(diallyldimethylammonium chloride) (PDDA), which is LbL-assembled with polyanion and clay as alternate anionic building blocks to form [polyelectrolyte1-polyelectrolyte2-polyelectrolyte1-clay]_n_-type multilayers. Our strategy was to incorporate a weak polyelectrolyte, poly(acrylic acid) (PAA), which is known to exhibit exponential growth, and laponite clay, a clay with relatively high charge density and small size. The multilayer thickness, composition, and structural organization were investigated, and we uncovered an additional molecular level contribution to the exponential growth: progressive increase in the degree of ionization of the system with multilayer build-up.

Based on our interesting findings, we further progressed to demonstrate a novel approach to obtain polymer-clay exponentially growing multilayers with high clay loading. We combined PAA and laponite as one deposition solution to form [PDDA-(Laponite + PAA)]_n._ multilayers. There have been prior studies on polyions being co-deposited with other similarly charged polyions [29], dyes [30], and surfactants [31], but to the best of our knowledge, this is the first work employing anionic clay and anionic polyelectrolyte for co-deposition as multilayers. The system displayed exponential growth with one of the highest reported clay loadings. We demonstrated a novel way of obtaining very thick multilayers composed of polyelectrolyte and clay, in which control over the composition and different physical properties could be achieved based on the initial mixing ratio of polyanion and clay. Our strategy can lead to new approaches to fabricate tailor-made nanomaterials for distinct applications.

## 2. Materials and Methods

**Figure A1:**
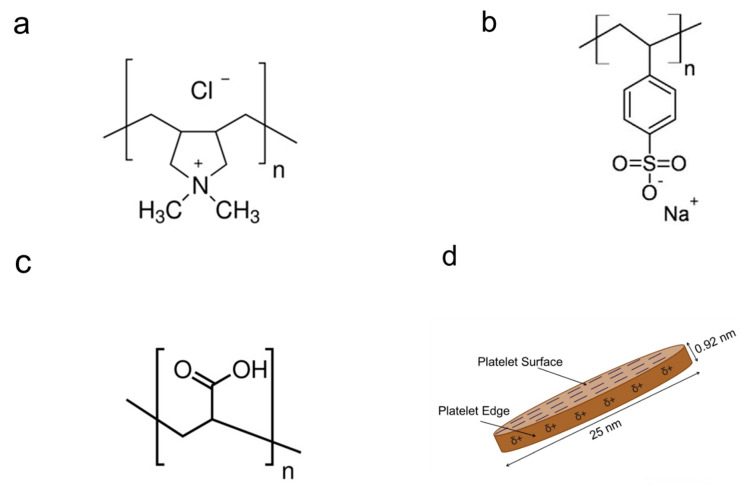
Chemical structure of (**a**) PDDA, (**b**) PSS, and (**c**) PAA. (**d**) Schematic of a laponite platelet.

**Materials:** Poly(diallyl dimethyl ammonium chloride) (PDDA) (MW 100,000–200,000) and poly(sodium-4-styrene sulfonate) (PSS) (MW ~70,000) were obtained from Sigma Aldrich, Inc. Poly(acrylic acid) (PAA) (MW ~340,000) was obtained from Polysciences Inc. For all experiments, 0.5 wt.% aqueous solutions of PDDA, PSS, and PAA were used unless otherwise noted. All solutions were prepared using Milli-Q water (18.2 MΩ cm) and were allowed to equilibrate for at least 12 h prior to use. The synthetic clay, laponite RD (empirical formula: Na_0.7_^+^ [(Si_8_Mg_5.5_Li_0.3_)O_20_(OH)_4_]^0.7−^), was purchased from Southern Clay Products, Inc. Laponite is a synthetic smectite and exists as stacks of platelets held together by intermittent sodium ions [32]. Dispersions of exfoliated laponite particles were used in all experiments. To obtain such dispersions, we followed a protocol similar to that of Podsiadlo et al. [33,34], where a clay dispersion of 0.5 wt.% is stirred vigorously for 5–7 days and allowed to sediment for a day, and then, the supernatant is obtained by decantation. The molecular structures of PDDA, PSS, and PAA and a schematic of a single laponite platelet are shown in Figure A1 (Appendix A).

**Substrate Cleaning/Charging:** The surfaces used as substrates for the LbL films were 25 mm × 75 mm quartz (Chemglass, Inc., Vineland, NJ, USA) or glass (Globe Scientific, Inc., Mahwah, NJ, USA) slides. The substrates were treated with freshly prepared piranha solution (70:30, 98 % H_2_SO_4_ and 30% H_2_O_2_) for one hour. This treatment exposes the free silanol (-Si-OH) groups on the substrate surface, which subsequently deprotonate, resulting in an overall negatively charged surface. The piranha solution treatment was followed by thorough rinsing with DI water and drying with air.

**Preparation of Multilayers**: For PDDA-Laponite, PDDA-PAA, and PDDA-PSS multilayers, the substrate was immersed in PDDA solution for 5 min, rinsed with DI water for 2 min, immersed in laponite dispersion (or PAA or PSS solutions for PDDA-PAA and PDDA-PSS systems, respectively) for 5 min, rinsed with DI water for 2 min, and dried with air for 1 min to form a bilayer. This procedure was repeated to form multilayers. A similar process was followed for the formation of PDDA-PAA-PDDA-Laponite and PDDA-PSS-PDDA-Laponite multilayers by using the corresponding polymer solutions or clay dispersions. In this work, the number of layers in the multilayer films corresponds to the number of individual deposition steps (e.g., a 140-layer PDDA-PAA film comprises 70 layers of PDDA and 70 layers of PAA).

**UV–Vis Spectroscopy:** The growth in multilayers was monitored by UV–Vis absorbance spectroscopy using a Hitachi U-1800 UV–Vis spectrophotometer. The calibration curves of the UV active components were obtained by spectroscopic analysis of their solutions. To record the absorbance of the multilayers, slides were placed in a specific location in front of the holder such that there was consistency in the region from where the spectrum was obtained. The absorbance recorded was an average of four readings, two from each side of the slide. Spectra were recorded in the range 190 nm–400 nm, sufficiently above and below the absorbance peaks of the components, i.e., 209 nm for PAA, 226 nm for PSS, and 245 nm for Laponite.

**Fourier Transform Infrared (FTIR) spectroscopy:** FTIR spectra were acquired using a Bruker Optics Vertex 70 FTIR spectrophotometer. The spectra were collected at a resolution of 2 cm^−1^ and as an average of 100 scans. The background for all measurements was air on the ZnSe plate. The multilayers were air-dried and scraped off the substrate to conduct the FTIR evaluation. FTIR spectra were used to estimate the absorbance intensities of chemical bonds/groups of interest, viz., the COO^−^ and COOH groups of PAA in the films.

**Scanning Electron Microscopy (SEM):** SEM images used to determine multilayer thickness and image the surface were obtained using a Hitachi SU-70 Scanning Electron Microscope at 20 kV. All samples were carbon-coated prior to imaging. To obtain cross-section images, the multilayers were immersed in liquid nitrogen before the slide was cut using a diamond cutter.

**Atomic Force Microscopy (AFM):** AFM imaging in tapping mode was performed on an Asylum Research MFP-3D AFM instrument. The measurements were conducted in air at ambient conditions by using Si cantilevers with a spring constant of ~42 N/m and a resonance frequency of about 300 kHz. AFM tips with a tip radius smaller than 10 nm were purchased from Olympus. The scan rate was 0.5–1.0 Hz. The scan lines and scan points were 512 each.

**X-Ray Diffraction (XRD):** Information about the layer spacing in the multilayers was obtained from XRD patterns using a Rigaku Ultima IV diffractometer (reflection geometry) with Cu K_α_ radiation (λ = 0.154 nm). The scan step was 0.05°, and the can speed was 0.35°/min. A small section of the glass slide with the deposited multilayers was cut to the size of the holder of the XRD instrument.

**Thermogravimetric Analysis (TGA):** TGA was carried out using NETZSCH TG209F1 Analyzer. The temperature increase rate was 10 °C/min, and the air flow rate was 20 mL/min.

## 3. Results and Discussion

### 3.1. LbL Assembly Using Polyelectrolyte and Clay Alternately as Anionic Deposition Solution: Polycation-Polyanion-Polycation-Clay

#### 3.1.1. Structural Comparison between Polycation-Clay and Polycation-Polyanion-Polycation-Clay Systems

We investigated the factors contributing to the assembly build-up, multilayer composition, layer spacing, and surface topography and determined the internal rearrangement and growth mechanism for the following multilayers: PDDA-Laponite, PDDA-PAA-PDDA-Laponite (with weak polyanion, PAA), and PDDA-PSS-PDDA-Laponite (with strong polyanion, PSS). A comparison of the three systems shed more light into the physicochemical aspects governing and structural organization and the multilayer growth mechanism.

**Figure 1:**
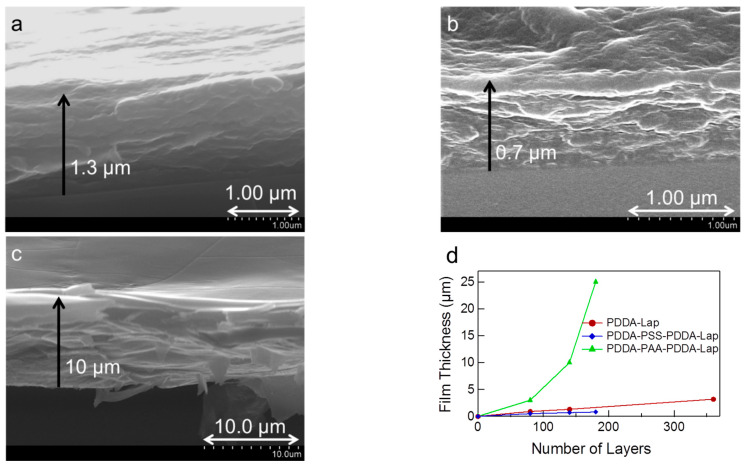
SEM cross-section images of 140-layer films of (**a**) PDDA-Laponite, (**b**) PDDA-PSS-PDDA-Laponite, and (**c**) PDDA-PAA-PDDA-Laponite. Lines with arrows indicate the thickness of the film deposited on the substrate. (**d**) Film thickness as a function of the number of layers for the three systems.

**Figure A2:**
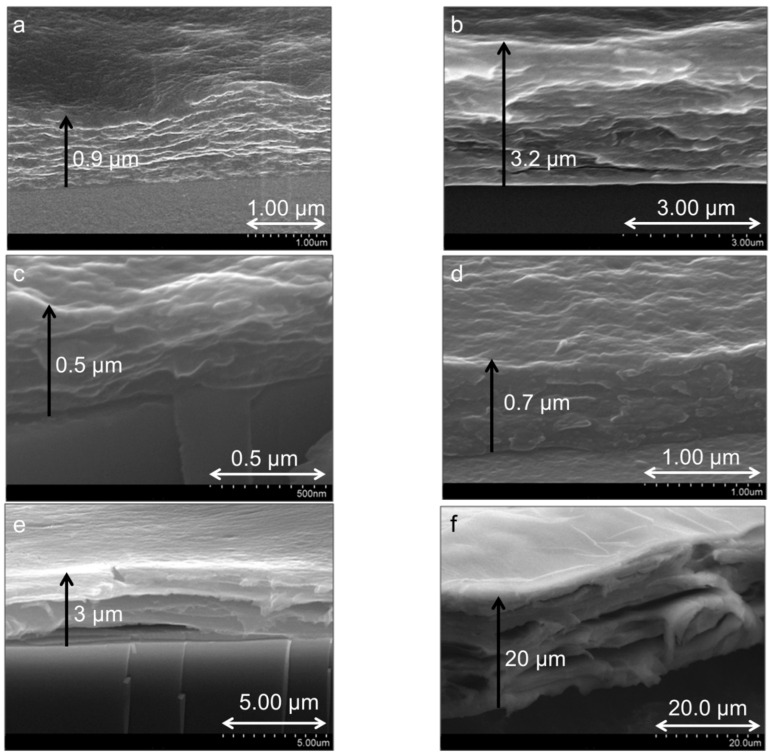
SEM cross-section images of (**a**) PDDA-Laponite 80 layers, (**b**) PDDA-Laponite 360 layers, (**c**) PDDA-PSS-PDDA-Laponite 80 layers, (**d**) PDDA-PSS-PDDA-Laponite 180 layers, (**e**) PDDA-PAA-PDDA-Laponite 80 layers, and (**f**) PDDA-PAA-PDDA-Laponite 180 layers. Lines with arrows indicate the thickness of the film deposited on the substrate.

**Figure A3:**
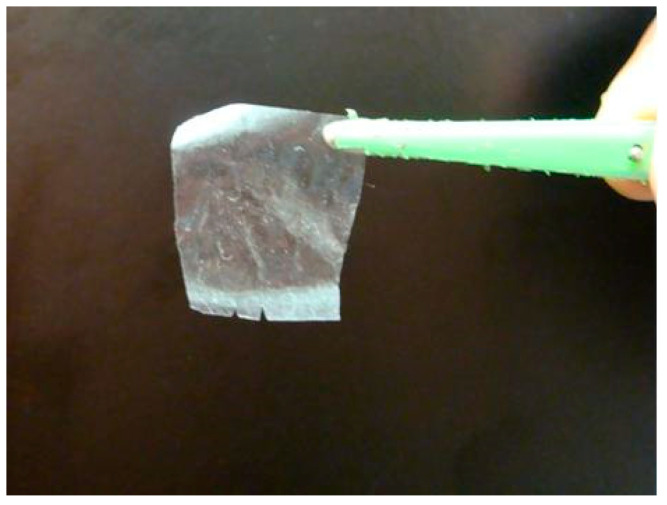
Photograph of a 180-layer PDDA-PAA-PDDA-Laponite free-standing film. (Size ~2 cm × 2 cm).

**Assembly Build-Up**: The film thickness of the different types of multilayers was determined by SEM cross-sectional images. The average film-thickness data for the various systems are summarized in Table 1. Representative SEM images are shown in Figure 1 and Figure A2 (Appendix A). The presence of stratified layers can be seen in the SEM image of PDDA-Laponite 140-layer film (Figure 1a). The SEM image of 140-layer PDDA-PSS-PDDA-Laponite multilayer demonstrates a thinner film (Figure 1b). The film thickness of PDDA-PAA-PDDA-Laponite multilayer for the same number of layers is an order of magnitude greater (Figure 1c). We note here that removal of multilayer films from water and exposure to air can cause some thinning, and exposure to vacuum during SEM can cause even more shrinking [35,36,37]. However, as discussed in the “Multilayer Composition” section of this work, the water content of PDDA-Laponite multilayers and PDDA-PSS-PDDA-Laponite multilayers is negligible based on TGA evaluation (Table 1). Thus, when these films are exposed to vacuum during SEM, the decrease of film thickness should be negligible. In the case of PDDA-PAA-PDDA-Laponite and PDDA-[Laponite + PAA] systems with water content between 12–21 wt.%, it is possible to have chain rearrangement and decrease in thickness as the multilayers are placed in vacuum, and hence, the reported film thickness may be slightly lower than the actual film thickness at ambient conditions. The relationship among the film thicknesses for different number of layers for the three systems by cross-sectional SEM images (Appendix A, Figure A2) is presented in Figure 1d. It can be seen that the thickness of the PDDA-Laponite (y = 0.009x, r^2^ = 0.99) and PDDA-PSS-PDDA-Laponite (y = 0.005x, r^2^ = 0.89) systems increases linearly with the deposition cycle, which is greater for the PDDA-Laponite system. The growth mechanism in PDDA-Laponite and PDDA-PSS-PDDA-Laponite is quite similar. PSS, a strong polyelectrolyte, is adsorbed on the surface during deposition just like the laponite particles. PSS molecules deposit in a stretched conformation, thus having less contribution to the thickness compared to individual layers of laponite, resulting in an overall thickness of the PDDA-PSS-PDDA-Laponite system that is smaller than that of PDDA-Laponite system for the same number of layers. Detailed studies of PDDA-PSS multilayer growth have revealed three different growth regimes: exponential, parabolic, and linear depending on the molecular weights of the polyelectrolytes used [38]. The three-component multilayer containing PAA, a weak polyelectrolyte, exhibits very large overall thickness, which increases exponentially with the number of layers, indicating a different growth mechanism. An exponential equation, y = 0.66 × 10^0.02x^ (r^2^ = 0.99), is a good fit for the increase in thickness in the PDDA-PAA-PDDA-Laponite multilayer. The PDDA-PAA-PDDA-Laponite film of 180 layers was very thick and clearly discernable on the substrate by the naked eye. It was also possible to separate/remove the film from the substrate to obtain a free-standing film (Appendix A, Figure A3). The film could be cut to desired dimensions using scissors.

**Figure 2:**
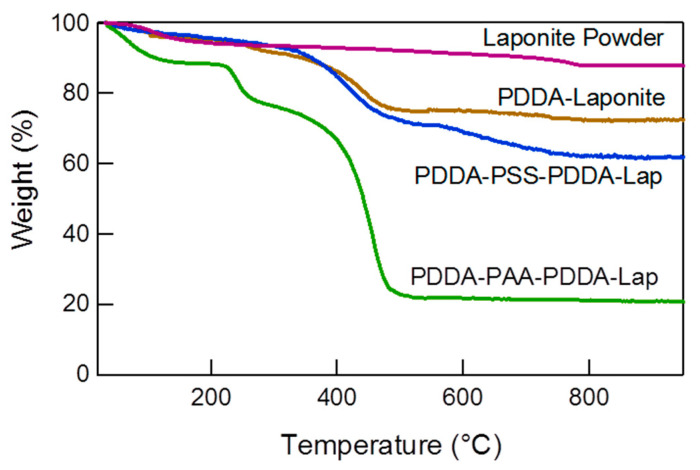
TGA thermograms of laponite powder and multilayers of PDDA-Laponite (360 layers), PDDA-PSS-PDDA-Laponite (180 layers), and PDDA-PAA-PDDA-Laponite (180 layers).

**Multilayer Composition:** The polyelectrolyte and clay contents of the different multilayers were determined by thermogravimetric analysis (TGA) (Figure 2), and their values are given in Table 1. The mass loss up to a temperature of 200 °C represents the water present on the surface and within the layers. It was determined that the water retention is higher for the PDDA-PAA-PDDA-Lap multilayer (~12 wt.%) compared to the PDDA-Laponite and PDDA-PSS-PDDA-Laponite, where the water content was negligible. This 12 wt.% water is strongly bound to the multilayer structure, similar to the “void water” as defined by Dodoo et al. [36,37]. In their work, they investigated the swelling behavior of PDADMAC-PSS multilayers prepared from aqueous solutions of 0.1 M or 0.5 M NaCl, and they found that the water content of the multilayers varied between ~40–60%, with the swelling being greater at higher salt concentrations. The authors stated that there are two types of water, the “void water” that fills the voids between the polymer chains and does not contribute to swelling, and the “swelling water”, which directly contributes to the swelling of the multilayers. For PDDA-Laponite multilayers, the laponite content was determined to be ~80 wt.%. Similar clay contents have been reported for poly(ethyleneimine)-Laponite [39], chitosan-montmorillonite [40], and polyacetylene-saponite [41] LbL assembled systems. The laponite content in PDDA-PSS-PDDA-Laponite multilayers was calculated to be ~50 wt.% and in PDDA-PAA-PDDA-Laponite multilayers ~22 wt.%. The difference in clay content between PSS-containing and PAA-containing multilayers is in agreement with a linear growth mechanism in the former and an exponential growth mechanism in the latter during their build-up. The interlayer diffusion of PAA would lead to very large amount of PDDA being adsorbed during the assembly build-up of PDDA-PAA-PDDA-Laponite multilayers [23]. The clay content in our exponentially growing system is much greater than the 5–10 wt.% reported by Podsiadlo et al. [26] for poly(ethyleneimine) (PEI), PAA, and montmorillonite clay multilayers (for similar number of layers and deposition times). On the contrary, the thickness of those multilayers was larger. A major factor contributing to the greater clay content could be the cyclic drying of the layers in our system. Exponential growth thrives in a gel-like multilayer structure where the interface between the deposition solution and bulk of the film is gradual [42]. Intermittent drying, in our case, would dehydrate the film and make it compact, orienting the clay particles along the surface and resulting in relatively smaller increments in thickness per cycle.

**Figure 3:**
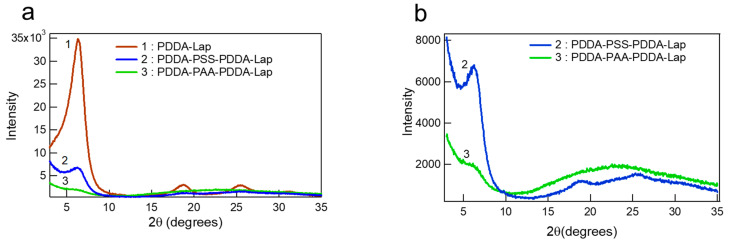
(**a**) XRD patterns of 360-layer PDDA-Laponite, 180-layer PDDA-PSS-PDDA-Laponite, and 180-layer PDDA-PAA-PDDA-Laponite multilayers. (**b**) The patterns of PDDA-PSS-PDDA-Laponite and PDDA-PAA-PDDA-Laponite multilayers are plotted at a smaller intensity range for better visualization of the peaks.

**Figure A4:**
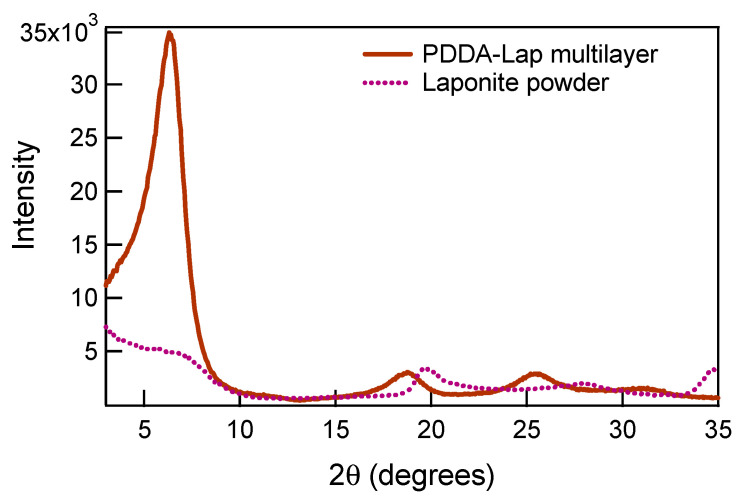
XRD patterns of laponite powder and PDDA-Laponite multilayers.

**Layer Spacing:** The layer spacing can be understood by the clay platelet arrangement with respect to each other. Figure 3 compares the XRD patterns of all three systems. The crystallographic properties are documented in Table 1. The PDDA-Laponite multilayers exhibit a very distinct and sharp (001) reflection peak at 2θ = 6.30°, corresponding to a basal spacing of 14.01 Å. Our results are in agreement with Kleinfeld et al. [13], who explained the basal spacing in PDDA-Laponite multilayers prepared by dripping LbL, as a combined contribution of PDDA (~5Å) and Laponite (~9Å) thickness. Similar basal spacing in spin-coated PDDA-Laponite multilayers has been reported [43]. PDDA-PSS-PDDA-Laponite multilayers demonstrated a fairly broad and very low intensity (001) peak almost six times less intense when compared to PDDA-Laponite multilayers. In the case of PDDA-PAA-PDDA-Laponite multilayers, a distinct (001) peak is not distinguishable. Similarly, the PDDA-Laponite multilayers exhibit a (003) reflection peak at 18.75° and a (004) reflection peak at 25.65°, the PDDA-PSS-PDDA-Laponite multilayers exhibit much less intense (003) peak at 18.55° and (004) at 25.30°, and the PDDA-PAA-PDDA-Laponite multilayers exhibit no (003) and (004) peaks. The difference in the intensities and nature of the peaks indicates that in PDDA-Laponite system, the laponite platelets orient almost parallel and equidistant to each other, whereas in the PSS- or PAA-containing systems, the clay platelets are not oriented parallel to the substrate, and there is a large variation in the amount of intercalated polymer. The similar structuring between PDDA-Laponite and PDDA-PSS-PDDA-Laponite multilayers suggests that the structuring is mainly formed by the clay platelets. The polyelectrolyte layers in the PSS-containing system are most likely deposited in an extended conformation, resulting in small increment in thickness. On the contrary, the clay platelets in the PAA-containing system are randomly oriented and unevenly spaced. The PAA molecules under the deposition conditions have low charge density and arrange in loopy conformations, resulting in greater deposition amount and irregular d spacing. Moreover, the internal rearrangement due to in-and-out diffusion could further contribute toward the irregular orientation of laponite particles [44]. The PDDA-PAA-PDDA-Laponite multilayer XRD pattern is similar in nature with the XRD pattern of laponite powder (Figure A4, Appendix A), where the clay platelets are randomly oriented.

**Figure 4:**
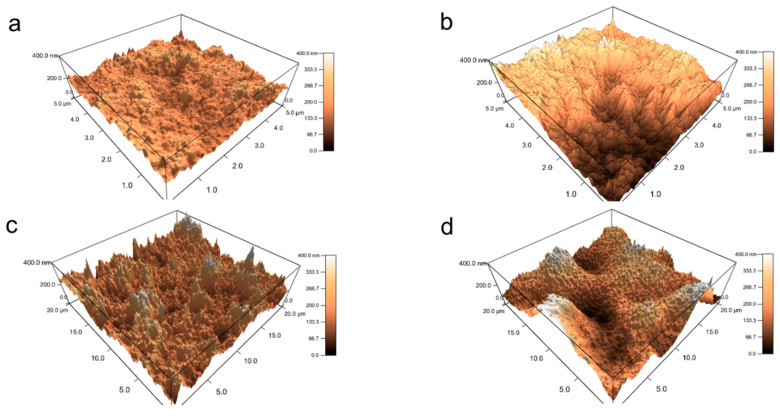
Representative AFM 3D height images of (**a**) PDDA-PSS-PDDA-Laponite 40 layers, surface roughness ~70 nm; (**b**) PDDA-PAA-PDDA-Laponite 40 layers, surface roughness ~145 nm; (**c**) PDDA-PSS-PDDA-Laponite 160 layers, surface roughness ~115 nm; and (**d**) PDDA-PAA-PDDA-Laponite 160 layers with fold-like surface topography, surface roughness ~560 nm. The scan size for (**a**,**b**) is 5 µm × 5 µm, and the scan size for (**c**,**d**) is 20 µm × 20 µm. All images are plotted for the same *z*-axis range of 400 nm.

**Figure A5:**
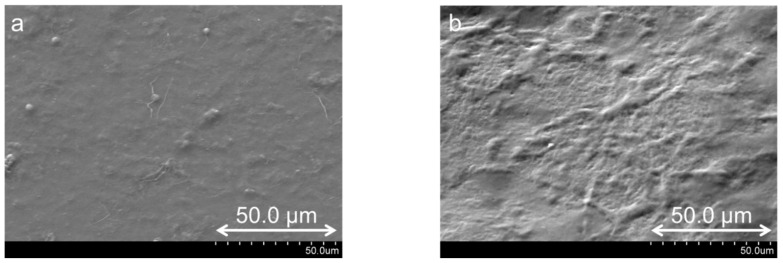
SEM images (top view) of (**a**) PDDA-PSS-PDDA-Laponite 160 layers and (**b**) PDDA-PAA-PDDA-Laponite 160 layers.

**Surface Topography**: The surface topography at different number of layers provides a perspective of internal arrangements as the assembly build-up takes place. Representative AFM 3D images of the outer surface of PDDA-PSS-PDDA-Laponite and PDDA-PAA-PDDA-Laponite multilayers are presented in Figure 4. Representative surface roughness values based on these images are reported in Table 1. For a particular number of layers, the PAA-containing multilayers appear rougher than the PSS-containing multilayers. Most notably, the AFM image in Figure 4d demonstrates that the PDDA-PAA-PDDA-Laponite film at 160 layers has large, fold-like features on the surface. Tjipto et al. [45] reported fold-like topography in multilayers and attributed that to the swelling and collapse of the film during adsorption and drying steps, respectively. SEM images at much lower magnifications (Figure A5, Appendix A) also show that the PDDA-PAA-PDDA-Laponite multilayers exhibit a rougher surface than the PDDA-PSS-PDDA-Laponite multilayers even at large scale (~100 µm × 100 µm). Ruths et al. [46] also attributed roughness to the “more than proportional” growth observed in PAH-PSS multilayer systems. Highly rough surface in PEI, PAA, and montmorillonite LbL films was also observed by Podsiadlo et al. [26] The increase in surface roughness is indicative of the disorderly increase in the number of PAA layers deposited. Moreover, the high surface area due to the fold-like surface topography can contribute to higher amounts being deposited with the increasing number of layers.

#### 3.1.2. Internal Reorganization and Multilayer Growth Mechanism in (PDDA-PAA-PDDA-Laponite)_n_

Based on the findings discussed in the previous sections, the total thickness of the films appears to be in the order: (PDDA-PAA-PDDA-Lap)_n_ > (PDDA-Lap)_2n_ > (PDDA-PSS-PDDA-Lap)_n_. The structural organization in PDDA-Laponite and PDDA-PSS-PDDA-Laponite multilayers is repeated as the multilayer build-up takes place; however, that in PDDA-PAA-PDDA-Laponite multilayers evolves as the build-up progresses. We can conclude that uniform/linear growth takes place in PDDA-Laponite and PDDA-PSS-PDDA-Laponite multilayers, whereas the PDDA-PAA-PDDA-Lap multilayers are governed by different physico-chemical phenomena, resulting in exponential growth. To better understand the growth mechanism in PDDA-PAA-PDDA-Laponite multilayers, the deposition amount, film optical properties, and ionic characteristics were monitored during the build-up.

**Figure 5:**
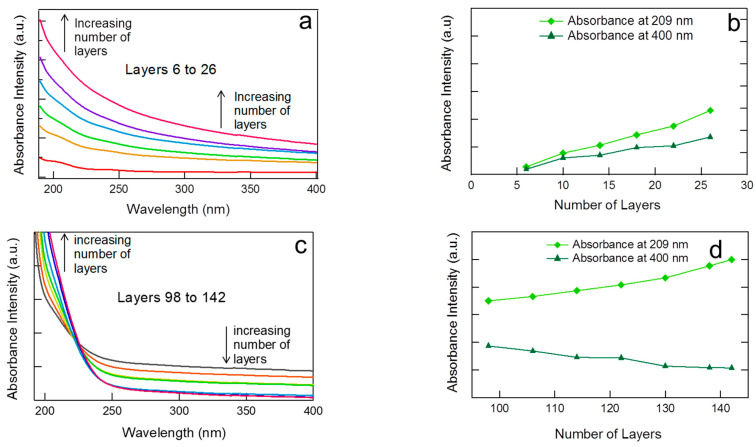
UV spectra of PDDA-PAA-PDDA-Laponite system; (**a**) UV spectra of multilayers for layers 6 to 26; (**b**) UV–Vis absorbance intensity at 209 nm (characteristic wavelength of PAA) and at 400 nm (visible light) as a function of the number of layers; (**c**) UV spectra of multilayers for layers 98 to 142; and (**d**) UV–Vis absorbance intensity at 209 nm and at 400 nm as a function of the number of layers.

**Layer Deposition Amount and Optical Characteristics:** The growth, in terms of deposition amount, the PDDA-PAA-PDDA-Laponite multilayers were monitored using UV–Vis absorbance spectroscopy (Figure 5). Data are presented after the sixth layer because it has been reported that in the first 4–6 layers, the substrate is not uniformly/totally covered, and during these initial layers, the layer build-up is not independent of the substrate effect [47,48]. PAA is UV active, and hence, the absorbance intensity at 209 nm, the characteristic wavelength of PAA, is proportional to the amount deposited. UV–Vis spectra of PDDA-PAA-PDDA-Laponite multilayers do not show a distinct peak at 209 nm because of the low absorptivity of PAA. The spectra shifted upwards with the increasing number of layers between 6–26 and are evenly spaced (Figure 5a). The absorbance intensity at 209 nm from Figure 5a plotted versus the number of layers exhibited a linear increasing trend, indicating that the mass of PAA deposited per cycle is almost constant at a lower number of layers (Figure 5b). For a higher number of layers, the 98th to 142nd layers, the overall spectra looked different than those of the lower numbers of layers. As it can be seen in Figure 5c, the absorbance intensity at 209 nm still increases, indicating that the amount of PAA deposited increases with the number of layers (Figure 5d).

The increasing trend observed for the absorbance intensity at 400 nm (visible light) indicates that the film formed on the glass substrate becomes opaque with increasing number of layers between layers 6 and 26 (Figure 5b). The UV–Vis spectroscopy of PDDA-PAA-PDDA-Laponite multilayer at a higher number of layers is quite different. At wavelengths below ~220 nm, the absorbance intensity is directly proportional to number of layers, whereas at wavelengths above ~220 nm, the intensity decreases with increasing number of layers (Figure 5c). This implies that at a higher number of layers, from 98–142, the assembly turns more and more transparent with the increase in the number of layers. The optical opacity of a polymeric multilayer film is attributed to heterogenous arrangement of the components in the assembly with defects in the internal arrangement [49]. Semicrystalline polymers are usually opaque, whereas amorphous polymers are transparent. In PDDA-PAA-PDDA-Laponite multilayers, the clay particles arrange randomly, as revealed by the XRD evaluation, hence the increase in transparency of the films.

**Figure 6:**
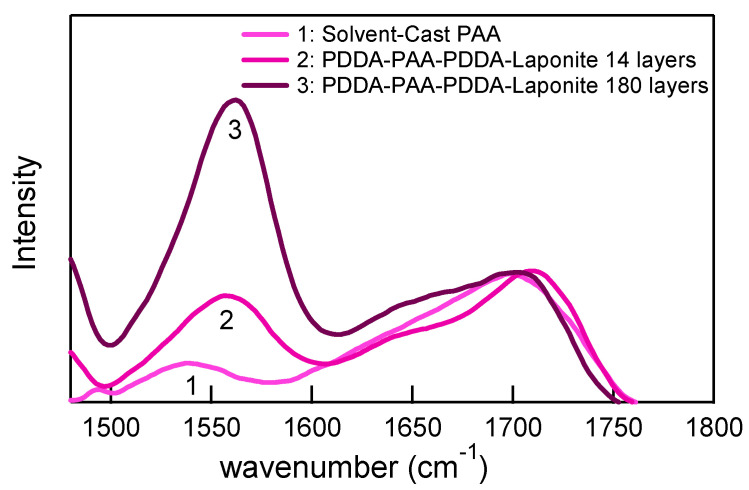
FTIR spectra of solvent-cast PAA and PDDA-PAA-PDDA-Laponite films of 14 layers and 180 layers. The peak at ~1700 cm^−1^ corresponds to –COOH groups, and the peak at ~1550 cm^−1^ corresponds to –COO^−^ groups.

**Ionic character of PAA:** The homogeneity in the film structure at a higher number of layers can be related to the increase in charge density of PAA in the multilayer structure. The comparison between FTIR spectra (Figure 6) of PAA cast from aqueous solution and two films of PDDA-PAA-PDDA-Laponite, a 14-layer and a 180-layer, showed that the ionization of PAA is greater at a higher number of layers. The peaks of consideration are at 1540–1550 cm^−1^ corresponding to –COO^−^ and at 1700–1710^−1^ corresponding to –COOH. PAA obtained from 0.5 wt.% solution exists mostly in its acid form with ~20% degree of ionization, whereas the 14-layer film has an almost equal ratio of acrylic acid peak and acrylate ion peak, i.e., ~50% degree of ionization. It has been reported that the degree of ionization of a weak polyacid increases in the charged environment of the multilayer structure [50,51]. Contradicting results in which PDDA-PAA multilayers exbibit similar degree of dissociation of the acrylic acid groups in films and in solution have also been reported [52]. We believe that in our system, the immersion of the PDDA-PAA-PDDA-Laponite multilayer into higher pH solutions (pH of 0.5 wt.% PDDA is ~4.5, pH of 0.5 wt.% Laponite is ~10.2, whereas pH of 0.5 wt.% PAA is ~3.3) during the LbL assembly process also contributes to the higher degree of ionization of PAA. Another interesting observation is that the degree of ionization is much greater for 180 layers, i.e., ~70% ionization. Thus, as the exponential growth progresses, the degree of ionization increases continuously. It has been reported that the average ionization of the carboxylic groups within the multilayer is suppressed when the multilayer was topped with a weak acid, and it was enhanced when the top layer was a polybase [53]. To the best of our knowledge, this is the first time a progressive upward shift in the degree of ionization observed during our multilayer build-up has been reported.

**Exponential Growth Mechanism**: Our findings uncover additional aspects of the “in-and-out diffusion” mechanism that is generally accepted as the mechanism of exponential growth in LbL assembly. During exponential growth, in our multilayers, there is a progressive increase in the charge density of the diffusing molecule that directly enhances the extent of electrostatic interaction, the driving force for the multilayer build-up. The increasing charge density during the build-up can also facilitate more water retention that results in a gel-like structure. Moreover, because of their random orientation, clay particles do not impede the “in-and-out diffusion”. The multilayer structure undergoes a swelling-collapsing cycle during the build-up, which creates fold-like surface morphology, resulting in greater surface area for deposition.

### 3.2. LbL Assembly Using Polyelectrolyte and Clay in Combination as a ‘Single’ Deposition Solution: (Polycation-(Polyanion + Clay))_n_

The two main findings on the internal dynamics of PDDA-PAA-PDDA-Laponite multilayers were the random orientation of the clay particles in the multilayer architecture and the progressive increase in the charge density of the system owing to the continuous increase in the degree of ionization of PAA. We further investigated another assembly approach comprising the same three building blocks but in which both PAA and Laponite are combined and deposited together in one step as the “polyanion” layer along with PDDA as the polycation. Polyelectrolyte and clay used together have been reported to exhibit synergistic structural effects [54]. Thus, this approach allows for the tailoring of the deposition composition and the potential to find conditions demonstrating synergism.

In this experiment, 0.5 wt.% aqueous solution of PDDA was used as the polycation solution, and a combination of 0.5 wt.% PAA and 0.5 wt.% Laponite aqueous solutions was used as the polyanion solution, resulting in PDDA-[Laponite + PAA] multilayers. Laponite and PAA, when combined in solution, resulted in a clear solution, indicating that the two were completely soluble in solution or interacted to only form nanoscale colloids. Four different weight ratios of Laponite and PAA solutions were used, viz., 4:1, 3:2, 2:3, and 1:4 and henceforth referred to as PDDA-[Laponite + PAA, 4:1], PDDA-[Laponite + PAA, 3:1], PDDA-[Laponite + PAA, 2:3], and PDDA-[Laponite + PAA, 1:4], respectively.

**Figure 7:**
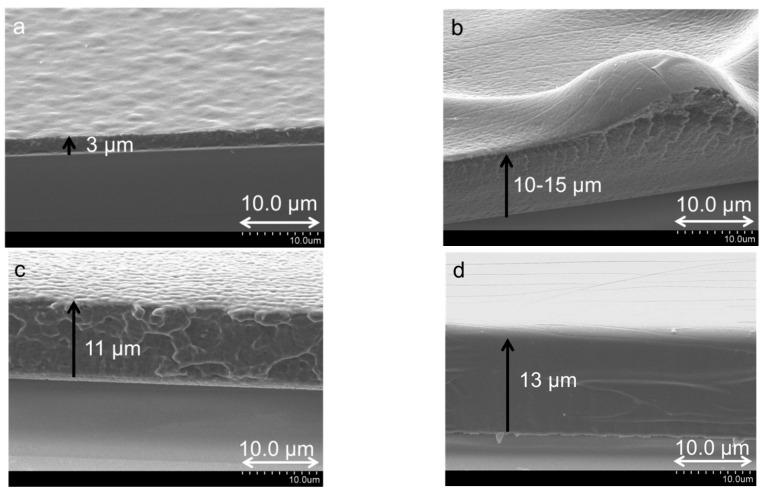
SEM cross-section images of 100 layers of (**a**) PDDA-[Laponite + PAA, 4:1], (**b**) PDDA-[Laponite + PAA, 3:2], (**c**) PDDA-[Laponite + PAA, 2:3], and (**d**) PDDA-[Laponite + PAA, 1:4] multilayers. Lines with arrows indicate the thickness of the film deposited on the substrate.

**Multilayer Thickness:** The thicknesses of 100-layered films of each of the PDDA-[Laponite + PAA] systems were obtained from SEM cross-section images (Figure 7) and are reported in Table 2. The thickness of PDDA-[Laponite + PAA, 4:1] multilayers was the lowest at ~3 µm (Figure 7a), while it varied between 10–15 µm (Figure 7b–d) for the other three systems, comparable to the thickness of the exponentially growing system, PDDA-PAA-PDDA-Lap multilayer (Table 1). Thicker layers are obtained for systems containing higher amounts of PAA, which is indicative of exponential growth driven by weak polyelectrolyte PAA.

Another factor that can influence the film thickness is the pH value of the starting solution. The pH of 0.5 wt.% PAA is 3.3, whereas the pH of 0.5 wt.% laponite dispersion is 10.2. The effect of clay solution pH on multilayer growth has been considered in polyethyleneimine/polyacrylic acid/polyethyleneimine/montmorillonite multilayers where the growth was reduced with decreasing montmorillonite pH primarily because of the increase in charge density of polyethyleneimine [55]. In our case, the pH of Laponite + PAA solution influences the charge density of the PAA. The pH values of the Laponite + PAA solutions were 7.4, 6.0, 5.0, and 4.4 for ratios of Laponite:PAA equal to 4:1, 3:2, 2:3, and 1:4, respectively. It is well-known that for a weak acid such as PAA, the degree of ionization is a function of the pH of the solution. The charge density influences the film thickness such that at higher charge density, PAA molecules deposit in extended conformation, and the amount deposited is less.

**Figure 8:**
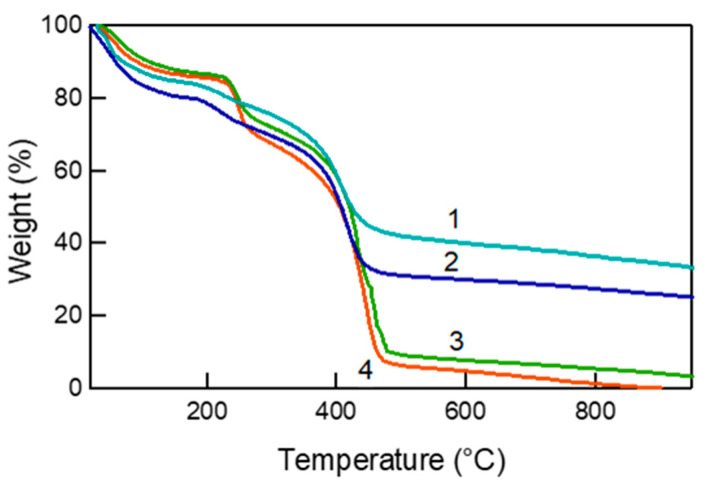
Thermogravimetric analysis of 100 layers of (1) PDDA-[Laponite + PAA, 4:1] multilayers, (2) PDDA-[Laponite + PAA, 3:2] multilayers, (3) PDDA-[Laponite + PAA, 2:3] multilayers, and (4) PDDA-[Laponite + PAA, 1:4] multilayers.

**Multilayer Composition**: The clay content of the films was determined by thermogravimetric analysis (Figure 8), and the values are presented in Table 2. The film composition supports the notion that a high proportion of PAA in the anion solution leads to greater amount of polymer deposited. The very low amount of Laponite clay in PDDA-[Laponite + PAA, 2:3] and PDDA-[Laponite + PAA, 1:4] multilayers is not only due to the lower content of clay in the starting solution but can also be attributed to the inability of clay to form LbL assembly at lower pH values. We have found that as the pH of Laponite is reduced from its natural pH, its zeta potential becomes less negative, and the hydrodynamic radius increases significantly, especially when the pH is reduced to 6.0 [56]. It has also been mentioned by van Duffel et al. [57] that clay particles, under very acidic conditions, can undergo chemical breakdown. We believe that for Laponite + PAA systems with ratio 2:3 and 1:4 where the pH is 5.0 and 4.4, respectively, the laponite particles may have aggregated due to loss of charge, and as a result, only a very small amount gets deposited. Thus, it is the PDDA and PAA that primarily contribute to the multilayer structure. A standout system appears to be the PDDA-[Laponite + PAA, 3:2], in which the clay content is ~25 wt.%, and the film thickness is very large. This finding most likely is a result of synergism between PAA and laponite. The pH conditions are such that are suitable for laponite dispersion in solution as individual platelets and for PAA to deposit in large amounts. Interestingly, this condition also results in the highest water content observed (21 wt.%, Table 2), further supporting internal diffusion. The higher water content could be the reason for bulge-like features observed on SEM section view (Figure 7b), where the bulge region appears to be less dense than the remainder of the film. In this system, the PAA retains water, and the clay provides a physical barrier that prevents the water from escaping. PDDA-[Laponite + PAA, 4:1] multilayers exhibited the highest clay content of 35 wt.%. Again, the higher proportion of clay in the solution increases the likelihood of more clay being deposited. Moreover, at pH 7.4, PAA has a high charge density; thus, fewer molecules and in an extended conformation are deposited. The combined effect of high charge density of PAA and high clay loading would inhibit internal diffusion to a large extent and can lead to a growth mechanism similar to linear growth, resulting in relatively thin layers.

**Figure 9:**
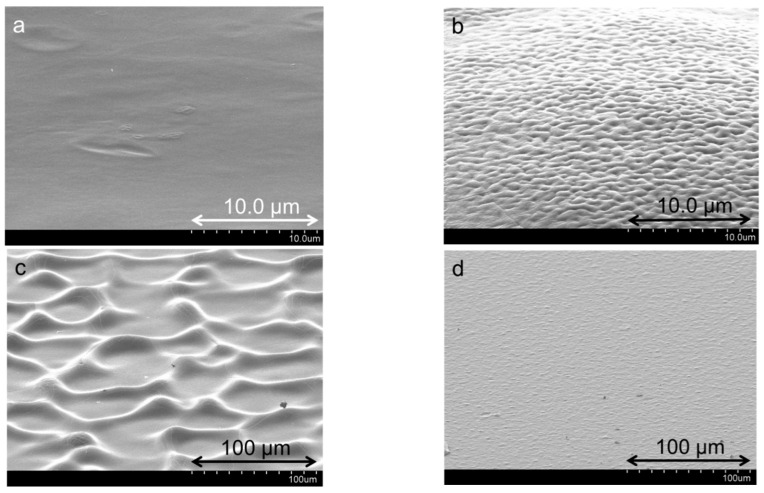
SEM surface images of 100 layers of (**a**) PDDA-[Laponite + PAA, 1:4], (**b**) PDDA-[Laponite + PAA, 2:3], (**c**) PDDA-[Laponite + PAA, 3:2], and (**d**) PDDA-[Laponite + PAA, 4:1] multilayers.

**Surface Topography**: The surface topography of the PDDA-[Laponite + PAA] multilayers at different ratios of laponite and PAA were also distinct. Figure 9a,b depicts the surface of PDDA-[Laponite + PAA, 1:4] and PDDA-[Laponite + PAA, 2:3] by SEM at a magnification of 5000×. Figure 9c,d depicts the surface for PDDA-[Laponite + PAA, 3:2] and PDDA-[Laponite + PAA, 4:1] at a 500× magnification. For the PDDA-[Laponite + PAA, 3:2] multilayer, large bulge-like features can be visualized most likely because of greater water retention as explained above. The other three systems also display different surface features because of different composition and growth mechanisms.

Overall, the results illustrate that the combination of PAA and laponite as one anionic solution for LbL deposition is a potential approach to achieve large growth in multilayer films incorporating clay. Moreover, this approach enables fabrication of films with tunable film thickness, surface topography, and composition. Understanding the possible interaction between PAA and laponite in solution (both are negatively charged, but laponite contains hydroxyl groups, and the platelets carry some positive charges in their edges, which can potentially interact with PAA) and the competition between the two towards LbL deposition has not been explored in this work and warrants further research. This knowledge would enable a greater degree of control over the film properties and can serve to predict suitable conditions to construct films for desired applications.

## 4. Conclusions

There is a strong demand for multicomponent materials, such as thin films incorporating polymer and clay, in many sectors of industry. LbL assembly is an inexpensive, facile, and versatile technique that holds great promise but faces the challenges of the speed of fabrication and precise control over properties. The speed of fabrication in polyelectrolyte-clay multilayers has been shown to increase by the use of electric field [58], but electrophoretic deposition takes away from the simplicity and versatility of the process. Fast build-up using dip-coating has been achieved by exponential growth but at the expense of clay content [26]. Knowledge gaps regarding the governing mechanism of exponential growth in polymer-clay systems exist [27,28], and researchers have pointed out the need for detailed studies [59,60].

In this work, enormous growth was achieved when poly(diallyldimethylammonium chloride) (PDDA) was LbL-assembled with poly(acrylic acid) (PAA) and laponite clay as alternating anionic building blocks. An unpredictable progressive increase in the degree of ionization of PAA contributing to exponential growth was uncovered. Additionally, the increasing charge density during build-up was found to facilitate more water retention, which in turn results in a gel-like structure of the multilayer. The progressive enhancement of the ionic environment and its resulting gel-like structure contribute to the interdiffusion of molecules towards homogeneity. Moreover, due to their random orientation, clay particles do not impede the “in-and-out diffusion”. The multilayer structure undergoes a swelling-collapsing cycle during build-up, which creates fold-like surface morphology resulting in greater surface area, an additional aspect for increased layer deposition.

These learnings were utilized to develop a novel method for achieving films with high clay loading as well as allowed for tailoring of the properties. The same building blocks were used, but PAA and laponite were combined as one anionic deposition solution. Films containing up to 25 wt.% clay could be obtained based on the synergistic combination of PAA and laponite. Moreover, the thickness, surface topography, and clay content could be manipulated by varying the mixing ratio of laponite and PAA, resulting in tailored multilayered films. Our findings shed new light on the mechanism of multilayer assembly of polyelectrolytes and clays. The results of our investigation hold potential for improving the efficacy of LbL assembly to fabricate previously unachievable polyelectrolyte-clay multilayer films.

## Figures and Tables

**Table 1:** Comparison of structural properties among the three clay-containing multilayers.

	Multilayers	PDDA-Laponite	PDDA-PSS-PDDA-Laponite	PDDA-PAA-PDDA-Laponite
Properties				
Thickness	80 layers	0.9 µm	0.5 µm	3 µm
	140 layers	1.3 µm	0.7 µm	10 µm
	180 layers	-	0.7 µm	20 µm
	360 layers	3.2 µm	-	-
Composition	Clay content	80 wt.%	50 wt.%	22 wt.%
	Water content	Negligible	Negligible	12 wt.%
Crystallographic properties	2θ	6.30° (Sharp peak)	6.25° (broad and low intensity peak)	No peak, only shoulder
	d Spacing	1.40 nm	1.41 nm	-
Surface roughness	40 layers	-	70 nm	145 nm
	160 layers	-	115 nm	560 nm

**Table 2:** Comparison of structural properties among the four systems in which laponite and PAA were combined in solution and deposited together for the multilayer build-up.

	Multilayers	PDDA-[Laponite + PAA]			
		Laponite: PAA (Weight Ratio of Aqueous Lap and PAA Solutions)			
Properties		4:1	3:2	2:3	1:4
Thickness	100 layers	~3 µm	10–15 µm	11 µm	13 µm
Composition	Clay content	35 wt.%	25 wt.%	5 wt.%	Negligible
	Water content	17 wt.%	21 wt.%	13 wt.%	14 wt.%

## Data Availability

Data supporting the reported results can be obtained by contacting Prof. Marina Tsianou.
